# Neuroprotective Effects of *Taraxacum officinale* Wigg. Extract on Glutamate-Induced Oxidative Stress in HT22 Cells via HO-1/Nrf2 Pathways

**DOI:** 10.3390/nu10070926

**Published:** 2018-07-19

**Authors:** Shan Huang, Ning Meng, Zhiming Liu, Li Guo, Linsha Dong, Bin Li, Qiang Ye

**Affiliations:** 1 Department of Pharmacy, Qingdao University of Science & Technology, Qingdao 266042, China; huangshan@qust.edu.cn (S.H.); 18354219361@163.com (N.M.); lzmqust@126.com (Z.L.); 15689483628@sina.cn (L.D.); leebin009@163.com (B.L.); 2 The College of Pharmacy, Chengdu University of Traditional Chinese Medicine, Chengdu 611137, China; Gli64@sina.com; 3 The Breeding Base of State Key Laboratory of Resources systems Research and Development Utilization of Chinese Herbal Medicines Constructioned by The Ministry of Science and Technology of the PRC and Sichuan Province, Chengdu 611137, China

**Keywords:** *Taraxacum officinale* Wigg., glutamate, oxidative stress, Nrf2/HO

## Abstract

Oxidative stress-mediated neuron damage is considered an important contributor to the pathogenesis and development of neurodegenerative diseases. *Taraxacum officinale* has been reported to possess antioxidant activities. However, whether it can protect neurons against oxidative damage and the underlying molecular mechanisms have not been fully determined. In the present study, we examined the neuroprotective effects of ethanol extracts of this plant (ETOW) on glutamate-induced oxidative stress in HT22 cells. Both cell viability and reactive oxygen species (ROS) assays showed that ETOW effectively attenuated glutamate-induced cytotoxicity and ROS generation. Furthermore, our results revealed that ETOW increased the expression of heme oxygenase-1 (HO-1) and promoted the nuclear translocation of nuclear factor erythroid 2-related factor-2 (Nrf2). The inhibitory effects of ETOW on glutamate-stimulated cell toxicity and ROS production were partially reversed by tin protoporphyrin (SnPP), an HO activity inhibitor. Taken together, these results demonstrate that ETOW can protect HT22 cells against glutamate-induced oxidative damage by inducing the Nrf2/HO-1 pathways. Our study supports the idea that *Taraxacum officinale* Wigg. is a promising agent for preventing neurodegenerative diseases.

## 1. Introduction

Oxidative stress (OS), a pathological metabolic condition arising from imbalance between generation and clearance of active reactive oxygen species (ROS) and reactive nitrogen species (RNS), can induce significant cell damage [1,2]. The central nervous system (CNS), with high polyunsaturated fatty acid content, high oxygen consumption, and weak antioxidative systems, is particularly vulnerable to OS, which causes neurodegenerative disorders [3,4,5]. Neurodegenerative diseases, including Alzheimer’s disease (AD), Parkinson’s disease (PD), amyotrophic lateral sclerosis (ALS), Huntington’s disease (HD) and Friedreich ataxia (FA), cause dementia, movement disorders, or motor disorders [1,6,7]. Several lines of evidence from the results of clinical and preclinical studies have suggested the presence of elevated levels of OS biomarkers and impairments to antioxidant defenses in the brain and peripheral tissues in neurodegenerative diseases [8]. Therefore, OS could serve as a potential treatment target, and therapeutic efforts have been aimed at reducing the ROS and strengthening the antioxidant defenses to prevent or alleviate neurodegenerative diseases.

Neural tissues are born with a panoply of antioxidant enzymes that work together with low molecular weight-free radical scavengers to reduce the cell damage caused by OS [9]. Deduced from in vitro and in vivo studies, heme oxygenases (HOs) have been recognized as dynamic sensors of cellular OS and likely arbiters of tissue redox homeostasis [9,10]. HO-1 is exquisitely sensitive to induction by OS compared to other HO proteins. HO-1 resides within the endoplasmic reticulum (ER), where it functions in concert with NADPH cytochrome P450 reductase, to oxidize pro-oxidant heme to free ferrous iron, carbon monoxide (CO) and radical-scavenging bile pigments, such as biliverdin (BV) and bilirubin to provide cytoprotection [10,11,12]. Previous reports have indicated that HO-1 has neuroprotective action [13,14]. These studies suggest that agents that induce HO-1 expression can be used to protect brain cells against oxidative and neurodegenerative conditions.

A number of phytomedicines have been proven to be effective free radical scavengers, which could reduce ROS and are beneficial for neurodegenerative diseases. Current research has explored indigenous medicines to revive cells from oxidative-mediated injury [15]. The genus *Taraxacum*, widely distributed in the warm-temperate zones of the Northern Hemisphere, belongs to the family Asteraceae, subfamily Cichorioideae, tribe Lactuceae [16]. The species *Taraxacum officinale*, commonly known as dandelion which stems from the French word “dent de lion” (“lion’s tooth”) [17], is an edible folk herb [18]; traditionally, it has been used in herbal medicine to treat various disorders such as hepatitis, cirrhosis, gallbladder disorders, digestive complaints, and rheumatic diseases [19,20]. And it is reported that *Taraxacum officinale* also exhibits several biological activities, including anti-cancer, hepatoprotective effect, anti-inflammatory effects, anti-obesity effects and antidepressant effects [21,22,23,24,25]. The leaves, flowers, and roots of *T. officinale* (TO) are often eaten fresh or cooked in salads, sandwiches, and snacks and are also used to make honey, juice, coffee substitutes, wines and other drinks [26,27]. TO crude extract is shown to possess antioxidant properties in in vitro and in vivo studies [28,29,30,31]. Chi-Su Yoon et al. reported that *Taraxacum coreanum* Nakai protects against glutamate-induced neuro-cytotoxicity in HT22 cells [32]. However, there have been no studies concerning the effects of TO extract on glutamate-induced neurodegenerative disorders. In this study, we intended to investigate the neuroprotective effect of ethanol extracts of *Taraxacum officinale* Wigg. (ETOW) and the underlying mechanism by which ETOW protects against glutamate-mediated neurotoxicity.

## 2. Materials and Methods

### 2.1. Chemicals

Ethanol (EtOH), formic acid and methanol (MeOH) were purchased from Merck Co. (Darmstadt, Germany). Dulbecco’s modified Eagle’s medium (DMEM), foetal bovine serum (FBS), and other tissue culture reagents were purchased from Gibco BRL Co. (Grand Island, NY, USA). Cobalt protoporphyrin (CoPP), HO-1 inducer, and tin protoporphyrin (SnPP) were obtained from Porphyrin Products. All other chemicals were obtained from Sigma-Aldrich (St. Louis, MO, USA), unless otherwise indicated.

### 2.2. Preparation of Extract

*Taraxacum officinale* Wigg. were obtained from Yili, Sinkiang, in August 2017 and were identified by Professor Huang Shan in the Qingdao University of Science & Technology. The *Taraxacum officinale* Wigg. whole plants were ground and extracted with 70% EtOH (1:20 *w*/*v*) two times under reflux for 2 h After filtration, the extracts were concentrated and evaporated to dryness and the yield is 18.6%. The extracts were stored at −20 °C until used for further experiments.

### 2.3. Characterization of ETOW

ETOW were analysed by liquid chromatography mass spectrometry-ion trap-time-of-flight (LCMS-IT-TOF) (SHIMADZU, Kyoto, Japan). Chromatography analyses of ETOW were carried out using ACQUITY UPLC HSS T3 column (2.1 × 100 mm, 1.8 μm) (Waters, Milford, MA, USA) maintained at 40 °C. The mobile phase consisted of 0.1% (*v*/*v*) formic acid solution (A) and MeOH (B). The gradient program was performed as follows: 0–20 min, 5–95% B; 20–23 min, 95% B in 23 min at a flow rate of 0.3 mL/min. Detection was done at a wavelength of 246 nm. The sample injection volume was 2 μL. Mass spectral analyses were performed with an electrospray ionization source (ESI). ESI conditions were as follows: temperature: 200 °C; nitrogen gas flow: 1.5 L/min; nebulizer pressure for positive mode 4500 V; nebulizer pressure for negative mode: −3500 V. Full mass scan spectra were recorded in positive and negative ion mode over a *m*/*z* range of 100–1000 daltons for both MS and MS/MS.

### 2.4. Cell Culture

Mouse hippocampal HT22 cells were obtained from Dr. Inhee Mook (Seoul National University, Gwanak-gu, Korea). HT22 cells were cultured in DMEM medium supplemented with 10% heat-inactivated FBS, streptomycin (100 mg/mL), penicillin G (100 U/mL), and l-glutamine (2 mM) and incubated in a humidified atmosphere containing 95% air and 5% CO_2_ at 37 °C.

### 2.5. Cell Viability Assays

Cell viability was evaluated by a 3-[4,5-Dimethylthiazol-2-yl]-2,5-diphenyltetrazolium bromide (MTT) assay. Cells (2 × 10^4^ cells/well in 96-well plates) were incubated with MTT at a final concentration of 0.5 mg/mL for 4 h Dimethyl sulfoxide (DMSO) was added to dissolve dark blue formazan crystals formed in the viable cells. Optical density was measured at 490 nm. The optical density of formazan formed in the control (untreated) cells was considered as 100% cell viability.

### 2.6. Measurement of Reactive Oxygen Species Assay

The fluorescent probe 2′,7′-dichlorofluorescein diacetate (DCF-DA) was used to measure the intracellular generation of ROS. HT22 cells (2.5 × 10^4^ cells/well in 24-well plates) were treated with 5 mM glutamate in the presence or absence of ETOW or SnPP (50 μM), an inhibitor of HO activity, and incubated for 8 h After washing with phosphate-buffered saline (PBS), the cells were stained with 10 μM DCF-DA for 30 min in the dark. The cells were further washed with PBS and extracted using 1% Triton X-100 for 10 min at 37 °C. Fluorescence was recorded at an excitation wavelength of 490 nm and emission wavelength of 525 nm using a spectrofluorometer (Spectramax Gemini XS; Molecular Devices, Sunnyvale, San Jose, CA, USA), respectively.

### 2.7. Preparation of Cytosolic and Nuclear Fractions

HT22 cells were homogenized (1:20, *w*/*v*) with PER-Mammalian Protein Extraction buffer (Pierce Biotechnology, Rockford, IL, USA) containing freshly added protease inhibitor cocktail I (EMD Biosciences, San Diego, CA, USA) and 1 mM phenylmethylsulfonyl fluoride (PMSF). The cytosolic fraction of the cells was prepared by centrifugation. The cytoplasmic and nuclear extracts of HT22 cells were isolated with NE-PER nuclear and cytoplasmic extraction reagents (Pierce Biotechnology, Rockford, IL, USA) according to the manufacturer’s instructions. Protein concentration was determined using a bicinchoninic acid (BCA) protein assay kit (Thermo Scientific, Rockford, IL, USA).

### 2.8. Western Blot

The expression of various proteins was analysed by Western blot. HT22 cells were lysed in 20 mM Tris-HCl buffer (pH 7.4) containing a protease inhibitor mixture (0.1 mM PMSF, 5 mg/mL aprotinin, 5 mg/mL pepstatin A, and 1 mg/mL chymostatin). Protein concentration was determined using a BCA protein assay kit. Thirty micrograms of protein extracts from each sample were separated with 12% sodium dodecyl sulfate-polyacrylamide gel electrophoresis (SDS-PAGE) and then electrophoretically transferred onto a Hybond-enhanced chemiluminescence (ECL) nitrocellulose membrane (Bio-Rad Laboratories, Hercules, CA, USA). The membrane was blocked with 5% non-fat dry milk and sequentially incubated with primary antibody and horseradish peroxidase (HRP)-conjugated secondary antibody (Santa Cruz Biotechnology, Dallas, TX, USA). The protein bands were visualized by ECL detection (Amersham Pharmacia Biotech, Piscataway, NJ, USA) and quantified with an image analysis program (Image Gauge v3.12 software; Fujifilm, Tokyo, Japan).

### 2.9. RNA Extraction and Reverse-Transcription Polymerase Chain Reaction (RT-PCR)

Total RNA was extracted from harvested cells using the TRIzol System (Invitrogen, Carlsbad, CA, USA) following the manufacturer’s recommendations. Total RNA was reverse-transcribed using a High Capacity RNA-to-cDNA kit (Applied Biosystems, Carlsbad, CA, USA). The cDNA was then amplified using a SYBR Premix Ex Taq kit (TaKaRa Bio Inc., Shiga, Japan) and a StepOnePlus Real-Time PCR system (Applied Biosystems). The primer sequences were as follows: HO-1, forward 5′-ACAGAAGAGGCTAAGACCGC, reverse 5′-TGTCAGGTATCTCCCTCCATT and β-actin, forward 5′-AGCCATGTACGTAGCCATCC, reverse 5′-CTCTCAGCTGTGGTGGTGAA [13]. The results were obtained in 28–30 cycles of amplification. PCR products were separated by electrophoresis, visualized under UV light, and quantified by densitometric analysis using volume integration with Quantity One (Bio-Rad Laboratories, Hercules, CA, USA).

### 2.10. Immunofluorescence

For localization of Nrf-2, HT22 cells were grown on Lab-Tek II chamber slides. After treatment with ETOW, cells were fixed in formalin and permeabilized with cold acetone. The cells were probed with a nuclear factor-erythroid-2-related factor 2 (Nrf2) antibody and fluorescein isothiocynate (FITC)-labelled secondary antibody (Alexa Fluor 488; Invitrogen, Carlsbad, CA, USA). To visualize the nuclei, cells were treated with 4′,6-diamino-2-phenylindole (DAPI, 1 μg/mL) for 30 min, washed with PBS for 5 min, and treated with 50 μL VectaShield (Vector Laboratories, Burlingame, CA, USA). Stained cells were visualized and photographed using a Provis AX70 fluorescence microscope (Olympus Optical, Tokyo, Japan).

### 2.11. Statistical Analysis

Data are expressed as the mean ± standard deviation (SD) of at least three independent experiments. To compare three or more groups, one-way analysis of variance (ANOVA) followed by Dunnett’s test was used. Statistical analysis was performed with GraphPad Prism software, version 3.03 (GraphPad Software Inc., San Diego, CA, USA).

## 3. Results

### 3.1. Characterization of ETOW by LC/MS

It has been reported that *Taraxacum* species have many kinds of phenolic acids and flavonoid glycosides [33]. As shown in Table 1 and determined through LC/MS analysis, ETOW contain cis-Caftaric acid, trans-Coutaric acid, Ferulic acid, Esculetin, 5-*O*-Caffeoylquinic acid, Caffeic acid, Chicoric acid, Luteolin, 11β,13-dihydro-taraxinic acid, and Quercetin-3′,4′,7-trimethyl ether [33,34,35,36].

### 3.2. Effects of ETOW on Cell Viability

**Figure 1:**
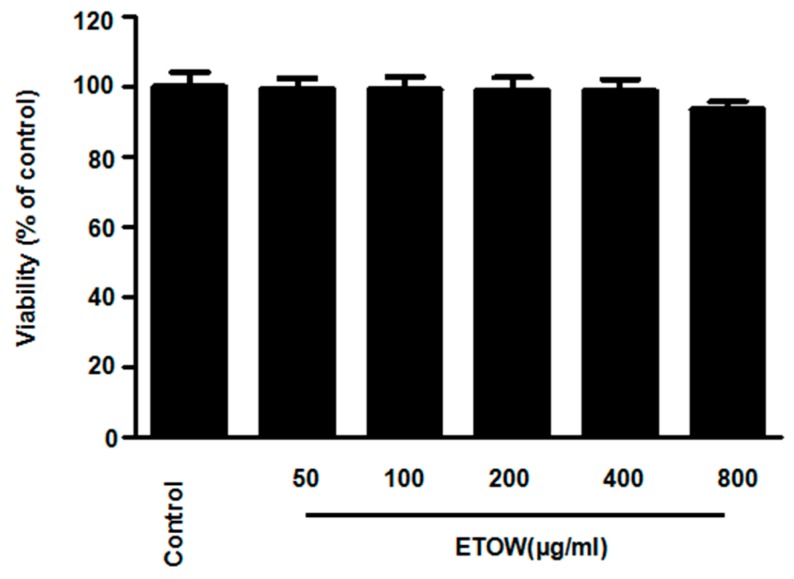
Effects of ETOW on cell viability in HT22 cells. HT22 cells were incubated for 12 h with various concentrations (50, 100, 200, 400, 800 μg/mL) of ETOW. Cell viability was determined by MTT. Each bar represents the mean ± SD (*n* = 3). ETOW, ethanol extracts of *Taraxacum officinale* Wigg.

To determine the cytotoxic potential of ETOW, the effects on mouse hippocampal HT22 cell viability was measured using the MTT assay. ETOW (50 to 400 µg/mL) did not show significant changes to cell viability but when increased to 800 µg/mL, the cell viability slightly decreased (Figure 1). Thus, the concentration range of ETOW was maintained between 50 and 400 µg/mL for subsequent experiments.

### 3.3. Effects of ETOW on Glutamate-Induced Cytotoxicity and Reactive Oxygen Species Production in HT22 Cells

**Figure 2:**
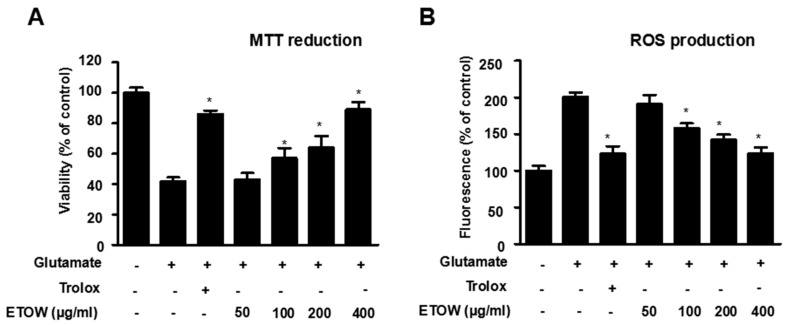
Effects of ETOW on glutamate-induced oxidative neurotoxicity and reactive oxygen species production in glutamate-induced HT22 cells. HT22 cells were pretreated with various concentrations (50, 100, 200, 400 µg/mL) of ETOW (**A**,**B**) and then incubated for 12 h with glutamate (5 mM). Trolox (50 μM) was used as a positive control. Cell viability (**A**) and ROS (**B**) production were measured by MTT assay and DCF fluorescence measurement, respectively. Values are calculated as percentages of untreated cells. Each bar represents the mean ± SD (*n* = 3). * *p* < 0.05 compared to the group treated with 5 mM glutamate. The “+”and “−” signs indicates the presence or absence.

To evaluate the neuroprotective effects of ETOW, the cytoprotective effects and ROS scavenging activities of ETOW were examined in glutamate-induced HT22 cells. After treatment with glutamate, the viability of HT22 cells significantly decreased compared to the untreated cells (Figure 2). Pretreatment with ETOW increased the cell viability of HT22 cell in a concentration-dependent manner (Figure 2A). Glutamate markedly increased intracellular ROS generation in HT22 cells. After pretreatment with ETOW, the generation of ROS induced by glutamate was downregulated significantly (Figure 2B). Trolox (50 μM), used as a positive control for its antioxidative effects, showed a significantly cytoprotective effect and ROS scavenging activity. The results demonstrated that ETOW attenuated glutamate-induced cytotoxicity and ROS generation in HT22 cells.

### 3.4. Effects of ETOW on Expression of HO-1 in HT22 Cells

**Figure 3:**
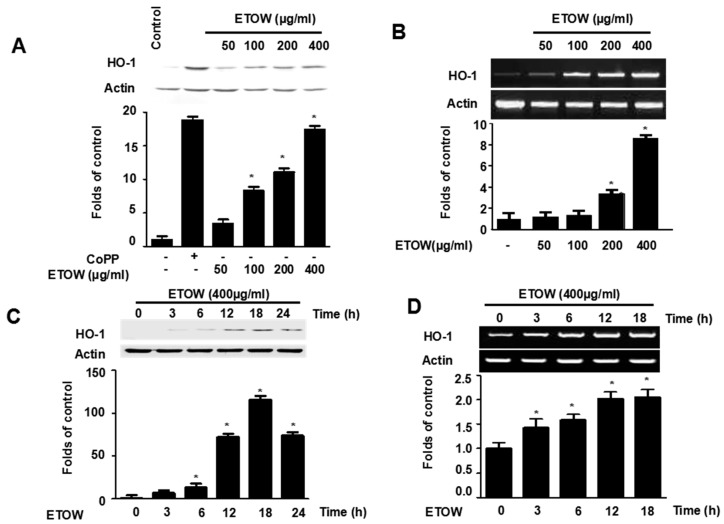
Effects of ETOW on HO-1 protein and mRNA expression in HT22 cells. HT22 cells were incubated for 12 h with various concentrations (50, 100, 200, 400 µg/mL) of ETOW (**A**,**B**). HT22 cells were incubated with 400 µg/mL of ETOW for various periods (**C**,**D**). CoPP (20 μM) was used as a positive control. The expression of HO-1 protein (A,C) and mRNA (B,D) were assessed by Western blot and RT-PCR, respectively. Protein and gene expression results for HO-1 were normalized to actin. Each bar represents the mean ± SD (*n* = 3). * *p* < 0.05 compared to the control group. The “+”and “−” signs indicates the presence or absence.

To determine the effect of ETOW on HO-1, the expression of HO-1 at both protein and mRNA levels was investigated following by ETOW treatment. ETOW increased the expression of HO-1 at both protein and mRNA levels in a concentration-dependent manner (Figure 3A,B). The HO-1 inducer CoPP, used as a positive control, significantly increased HO-1 expression at both protein and mRNA levels (Figure 3A,B). Treatment with ETOW (400 µg/mL) increased the protein and mRNA levels of HO-1 in a time-dependent manner within 18 h, and levels slightly decreased thereafter (Figure 3C,D). The results showed that ETOW promoted HO-1 expression.

### 3.5. Effects of ETOW on Nrf2 Translocation in HT22 Cells

**Figure 4:**
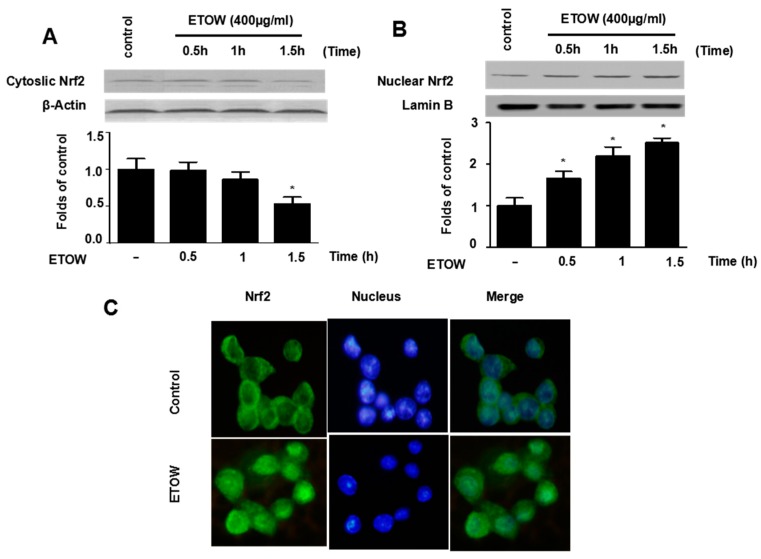
Effects of ETOW on Nrf2 translocation in HT22 cells. HT22 cells were incubated with 400 µg/mL of ETOW for 0.5, 1, or 1.5 h. The cytosolic and nuclear extracts were fractionated using PER-Mammalian Protein Extraction buffer. Nrf2 protein expression was detected by Western blot in cytosolic and nuclear extracts (**A**,**B**). Nrf2 translocation was detected by immunofluorescence (**C**). The levels of cytosolic and nuclear Nrf2 were normalized to the β-actin and lamin B, respectively. * *p* < 0.05.

Because Nrf2 is a key upstream modulator HO-1 induction [37], the Nrf2 translocation was investigated. After incubation with ETOW (400 µg/mL), the expression of Nrf2 in nuclear fractions was increased in a time-independent manner (Figure 4A). In contrast, the expression of Nrf2 in cytosolic fractions declined accordingly (Figure 4B). Similar results were observed using immunofluorescence microscopy (Figure 4C). The results indicated that ETOW induced Nrf2 translocation.

### 3.6. Effects of ETOW-Induced HO-1 Expression Pathway in Glutamate-Induced HT22 Cells

**Figure 5:**
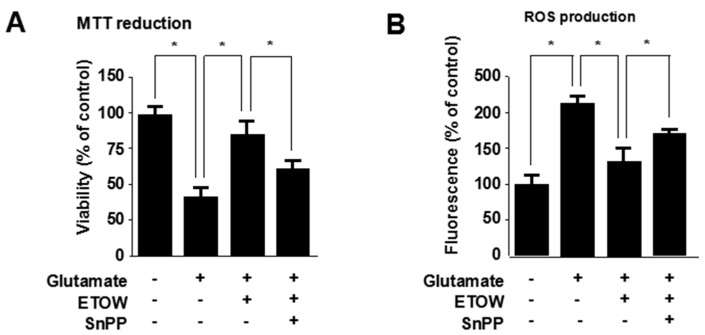
Effects of ETOW-induced HO-1 on glutamate-induced cytotoxicity and reactive oxygen species generation in HT22 cells. HT22 cells were pretreated with 400 µg/mL ETOW in the presence or absence of 50 μM SnPP and then exposed to glutamate (5 mM) for 12 h Cell viability (**A**) and ROS (**B**) production were measured by the MTT assay and DCF fluorescence measurement, respectively. Each bar represents the mean ± SD (*n* = 3). * *p* < 0.05. The “+”and “−” signs indicates the presence or absence.

To investigate the role of HO-1 in the neuroprotective effects of ETOW, the HO-1 pathway was inhibited by SnPP. ETOW significantly suppressed glutamate-induced cytotoxicity (Figure 5A) and ROS generation (Figure 5B). SnPP treatment effectively reversed the inhibitory action of ETOW on glutamate-induced cytotoxicity and reactive oxygen species generation (Figure 5A,B). Therefore, it could be deduced that ETOW inhibited glutamate-induced cytotoxicity and ROS generation in part by inducing the HO-1 expression pathway.

## 4. Discussion

Neurodegenerative diseases, a heterogeneous group of disorders characterized by gradually progressive, selective loss of anatomically or physiologically related neuronal systems, would result in the worsening of neurological dysfunction and are ultimately fatal [1,7,38]. It is necessary to diagnose timely and to conduct the appropriate therapeutic intervention to stop the progression of disease and suffering of patients. Traditional Chinese medicine has potential in the treatment of neurodegenerative diseases for its multi-target, multi-link, multi-way characteristics. For example, *Ginkgo biloba* [39], *Panax ginseng* [40], and *Astragalus* [41] have shown neuroprotective effects. In this work, we studied the neuroprotective activity of ETOW in glutamate-stimulated HT22 cells.

Glutamate is the major excitatory neurotransmitter in the mammalian central nervous system [42]. However, an excessive concentration of glutamic acid leads to death of neurons [43]. Neuron damage can be triggered by glutamate in two ways: One is glutamate receptor-mediated oxidative excitotoxicity caused by the excessive influx of Ca^2+^ and modification of calcium homeostasis; the other is ROS-linked oxidative damage which is mainly linked to the glutamate/cysteine anti-porter requiring delivery of cysteine [15]. The HT22 cells, an immortalized neuronal cell line derived from the mouse hippocampus, lack functional ionotropic glutamate receptors [44]. Therefore, the HT22 cells are frequently used for studying the non-receptor-mediated oxidative glutamate toxicity mechanism, namely, in the glutamate-induced OS in vitro model which excludes excitotoxicity as a cause for glutamate-stimulated neuronal death [45]. In our present study, we investigated the neuroprotective effects of *Taraxacum officinale* Wigg. in glutamate-induced HT22 cells. We found that non-toxic concentrations of ETOW were able to attenuate glutamate-induced cytotoxicity and ROS generation (Figure 2A,B), suggesting that ETOW could act as a potential neuroprotective agent.

HO-1, a 32 kDa member of the stress protein superfamily, is also known as heat shock protein (Hsp)-32. HO-1 and its enzymatic products including CO, BV and bilirubin, play important roles in regulating the biological oxidative stress system [14]. There is ample evidence implicating a neuroprotective role for HO-1 both in vitro and in vivo [10]. The nuclear transcriptional factor, Nrf2, which belongs to the Cap ‘N’ Collar (CNC) family that contains a conserved basic leucine zipper (bZIP) transcription factor, plays an important role in HO-1 expression [46]. The Nrf2-medicated pathway is known as an essential protective mechanism against OS [47]. Under physiological conditions, Nrf2 remains in the cytoplasm bound to Kelch-like ECH associated protein 1 (Keap1), an Nrf2 inhibitor that acts as a sensor for ROS/electrophilic stress, and Bach1, a bZip transcription factor, forms heterodimers with musculoaponeurotic fibrosarcoma oncogene homologue K (MafK); under stressful conditions, Nrf2 releases from Keap1 and translocates to the nucleus where it heterodimerizes with a small Maf protein, binds to antioxidant response element (ARE), and finally activates HO-1 gene transcription [46,48,49,50]. In our investigation, we found that ETOW induced HO-1 protein expression and increased the levels of Nrf2 in the nucleus (Figure 3 and Figure 4). In addition, experiments with SnPP indicated that the expression of HO-1 evoked by ETOW was positively associated with neuroprotective effects in HT22 cells (Figure 5). These results indicated that ETOW might exert neuroprotective effects by activating Nrf2/HO-1.

Taken together, our study demonstrated that ETOW attenuated glutamate-induced OS in HT22 cells, which is related to activation of Nrf2/HO-1 (by promoting Nrf2 translocation to the nucleus and HO-1 expression). Therefore, *Taraxacum officinale* Wigg. may have the potential to be used for neuroprotective agents through suitable exploitation and uptake.

## Figures and Tables

**Table 1:** Composition of ETOW.

Tentative Identification	M	t_R_ (min)	*m*/*z* Traces(−)	*m*/*z* Traces(+)	MS^2^
cis-Caftaric acid	312	2.4–2.6	311.0283	335.0603	MS^2^(−)[311.0285]: 149.0075, 179, 135.0441
trans-Coutaric acid	296	3.7–3.8	295.0349		MS^2^(−)[295.0354]: 163.0370,119.0506, 113, 164
Ferulic acid	194	4.26–4.3		217.1082	MS^2^(+)[217.1082]: 217.1086, 144.0847
Esculetin	178	4.45–4.50	177.0147		
5-*O*-Caffeoylquinic acid	354	4.55–4.57	353.0755		MS^2^(−)[353.0750]: 191.051, 354.077, 179
Caffeic acid	180	4.6	179.0313		MS^2^(−)[179.0323]: 135
Chicoric acid	474	6.4–6.49	473.0541		MS^2^(−)[473.0563]: 311.029, 293, 179.033, 474.059, 149, 312, 294, 219.0252, 341.0544
Luteolin	286	10.3–10.4	285.0308	287.0739	
11β,13-dihydro-taraxinic acid	264	10.7	263.1202		MS^2^(−)[263.1202]: 219.1327, 245.1085
Quercetin-3’,4’,7-trimethyl ether	344	18.5–18.6		345	MS^2^(+)[345.2637]: 345.2638

ETOW, ethanol extracts of *Taraxacum officinale* Wigg.

## References

[1] Ienco E.C., Logerfo A., Carlesi C., Orsucci D., Ricci G., Mancuso M., Siciliano G. (2011). Oxidative stress treatment for clinical trials in neurodegenerative diseases. J. Alzheimers Dis..

[2] Barnham K.J., Masters C.L., Bush A.I. (2004). Neurodegenerative diseases and oxidative stress. Biomed. Pharmacother..

[3] Di Carlo M., Giacomazza D., Picone P., Nuzzo D., San Biagio P.L. (2012). Are oxidative stress and mitochondrial dysfunction the key players in the neurodegenerative diseases?. Free Radic. Res..

[4] Guerraaraiza C., Álvarezmejía A.L., Sáncheztorres S., Farfangarcía E., Mondragónlozano R., Pintoalmazán R., Salgadoceballos H. (2013). Effect of natural exogenous antioxidants on aging and on neurodegenerative diseases. Free Radic. Res..

[5] Li J., Wuliji O., Li W., Jiang Z.G., Ghanbari H.A. (2013). Oxidative Stress and Neurodegenerative Disorders. Int. J. Mol. Sci..

[6] Solanki I., Parihar P., Mansuri M.L., Parihar M.S. (2015). Flavonoid-Based Therapies in the Early Management of Neurodegenerative Diseases. Adv. Nutr..

[7] Peden A.H., Ironside J.W. (2012). Molecular pathology in neurodegenerative diseases. Curr. Drug Targets.

[8] Niedzielska E., Smaga I., Gawlik M., Moniczewski A., Stankowicz P., Pera J., Filip M. (2016). Oxidative Stress in Neurodegenerative Diseases. Mol. Neurobiol..

[9] Schipper H.M. (2004). Heme oxygenase expression in human central nervous system disorders. Free Radic. Biol. Med..

[10] Schipper Hyman M., Song W., Zukor H., Hascalovici Jacob R., Zeligman D. (2009). Heme oxygenase-1 and neurodegeneration: Expanding frontiers of engagement. J. Neurochem..

[11] Schipper H.M. (2011). Heme oxygenase-1 in Alzheimer disease: A tribute to Moussa Youdim. J. Neural Transm..

[12] Schipper Hyman M. (2006). Heme Oxygenase-1: Transducer of Pathological Brain Iron Sequestration under Oxidative Stress. Ann. N. Y. Acad. Sci..

[13] Chao X.J., Chen Z.W., Liu A.M., He X.X., Wang S.G., Wang Y.T., Liu P.Q., Ramassamy C., Mak S.H., Cui W. (2014). Effect of Tacrine-3-caffeic Acid, A Novel Multifunctional Anti-Alzheimer’s Dimer, Against Oxidative-Stress-Induced Cell Death in HT22 Hippocampal Neurons: Involvement of Nrf2/HO-1 Pathway. CNS Neurosci. Ther..

[14] Barone E., Di Domenico F., Mancuso C., Butterfield D.A. (2014). The Janus Face of the Heme Oxygenase/Biliverdin Reductase System in Alzheimer Disease: It’s Time for Reconciliation. Neurobiol. Dis..

[15] Akanda M.R., Kim M.-J., Kim I.-S., Ahn D., Tae H.-J., Rahman M.M., Park Y.-G., Seol J.-W., Nam H.-H., Choo B.-K. (2018). Neuroprotective Effects of Sigesbeckia pubescens Extract on Glutamate-Induced Oxidative Stress in HT22 Cells via Downregulation of MAPK/caspase-3 Pathways. Cell. Mol. Neurobiol..

[16] Schütz K., Carle R., Schieber A. (2006). Taraxacum—A review on its phytochemical and pharmacological profile. J. Ethnopharmacol..

[17] Esatbeyoglu T., Obermair B., Dorn T., Siems K., Rimbach G., Birringer M. (2017). Sesquiterpene Lactone Composition and Cellular Nrf2 Induction of Taraxacum officinale Leaves and Roots and Taraxinic Acid β-d-Glucopyranosyl Ester. J. Med. Food.

[18] González-Castejón M., García-Carrasco B., Fernández-Dacosta R., Dávalos A., Rodriguez-Casado A. (2013). Reduction of Adipogenesis and Lipid Accumulation by Taraxacum officinale (Dandelion) Extracts in 3T3L1 Adipocytes: An in vitro Study. Phytother. Res..

[19] Ivanov I., Petkova N., Tumbarski J., Dincheva I., Badjakov I., Denev P., Pavlov A. (2017). GC-MS characterization of n-hexane soluble fraction from dandelion (Taraxacum officinale Weber ex F.H. Wigg.) aerial parts and its antioxidant and antimicrobial properties. Z. Naturforsch. C.

[20] Davaatseren M., Hur H.J., Yang H.J., Hwang J.-T., Park J.H., Kim H.-J., Kim M.J., Kwon D.Y., Sung M.J. (2013). Taraxacum official (dandelion) leaf extract alleviates high-fat diet-induced nonalcoholic fatty liver. Food Chem. Toxicol..

[21] Yoon J.Y., Cho H.S., Lee J.J., Lee H.J., Jun S.Y., Lee J.H., Song H.H., Choi S., Saloura V., Park C.G. (2016). Novel TRAIL Sensitizer Taraxacum Officinale F.H. Wigg Enhances TRAIL-Induced Apoptosis in Huh7 Cells. Mol. Carcinog..

[22] Domitrović R., Jakovac H., Romić Ž., Rahelić D., Tadić Ž. (2010). Antifibrotic activity of Taraxacum officinale root in carbon tetrachloride-induced liver damage in mice. J. Ethnopharmacol..

[23] Koh Y.J., Cha D.S., Ko J.S., Park H.J., Choi H.D. (2010). Anti-inflammatory effect of Taraxacum officinale leaves on lipopolysaccharide-induced inflammatory responses in RAW 264.7 cells. J. Med. Food..

[24] Zhang J., Kang M.J., Kim M.J., Kim M.E., Song J.H., Lee Y.M., Kim J.I. (2008). Pancreatic lipase inhibitory activity of taraxacum officinale in vitro and in vivo. Nutr. Res. Pract..

[25] Li Y.C., Shen J.D., Li Y.Y., Huang Q. (2014). Antidepressant effects of the water extract from Taraxacum officinale leaves and roots in mice. Pharm. Biol..

[26] Martinez M., Poirrier P., Chamy R., Prüfer D., Schulze-Gronover C., Jorquera L., Ruiz G. (2015). Taraxacum officinale and related species—An ethnopharmacological review and its potential as a commercial medicinal plant. J. Ethnopharmacol..

[27] Hfaiedh M., Brahmi D., Zourgui L. (2014). Hepatoprotective effect of Taraxacum officinale leaf extract on sodium dichromate-induced liver injury in rats. Environ. Toxicol..

[28] Park C.M., Park J.Y., Noh K.H., Shin J.H., Song Y.S. (2011). Taraxacum officinale Weber extracts inhibit LPS-induced oxidative stress and nitric oxide production via the NF-κB modulation in RAW 264.7 cells. J. Ethnopharmacol..

[29] Colle D., Arantes L.P., Gubert P., Da L.S., Athayde M.L., Teixeira Rocha J.B., Soares F.A. (2012). Antioxidant properties of Taraxacum officinale leaf extract are involved in the protective effect against hepatoxicity induced by acetaminophen in mice. J. Med. Food.

[30] Colle D., Arantes L.P., Rauber R., de Mattos S.E.C., Rocha J.B.T.D., Nogueira C.W., Soares F.A.A. (2012). Antioxidant properties of Taraxacum officinale fruit extract are involved in the protective effect against cellular death induced by sodium nitroprusside in brain of rats. Pharm. Biol..

[31] Choi U.-K., Lee O.-H., Yim J.H., Cho C.-W., Rhee Y.K., Lim S.-I., Kim Y.-C. (2010). Hypolipidemic and Antioxidant Effects of Dandelion (Taraxacum officinale) Root and Leaf on Cholesterol-Fed Rabbits. Int. J. Mol. Sci..

[32] Yoon C.S., Ko W., Lee D.S., Kim D.C., Kim J., Choi M., Beom J.S., An R.B., Oh H., Kim Y.C. (2017). Taraxacum coreanum protects against glutamate-induced neurotoxicity through heme oxygenase-1 expression in mouse hippocampal HT22 cells. Mol. Med. Rep..

[33] Schütz K., Kammerer D.R., Carle R., Schieber A. (2005). Characterization of phenolic acids and flavonoids in dandelion (Taraxacum officinale WEB. ex WIGG.) root and herb by high-performance liquid chromatography/electrospray ionization mass spectrometry. Rapid Commun. Mass Spectrom..

[34] Shi S., Zhao Y., Zhou H., Zhang Y., Jiang X., Huang K. (2008). Identification of antioxidants from Taraxacum mongolicum by high-performance liquid chromatography–diode array detection–radical-scavenging detection–electrospray ionization mass spectrometry and nuclear magnetic resonance experiments. J. Chromatogr. A.

[35] Shi S.Y., Zhang Y.P., Zhou H.H., Huang K.L., Jiang X.Y. (2010). Screening and identification of radical scavengers from Neo-Taraxacum siphonanthum by online rapid screening method and nuclear magnetic resonance experiments. J. Immunoass. Immunochem..

[36] Mingarro D.M., Plaza A., Galán A., Vicente J.A., Martínez M.P., Acero N. (2015). The effect of five Taraxacum species on in vitro and in vivo antioxidant and antiproliferative activity. Food Funct..

[37] Li B., Jeong G.S., Kang D.G., Lee H.S., Kim Y.C. (2009). Cytoprotective effects of lindenenyl acetate isolated from Lindera strychnifolia on mouse hippocampal HT22 cells. Eur. J. Pharmacol..

[38] Lin M.T., Beal M.F. (2006). Mitochondrial dysfunction and oxidative stress in neurodegenerative diseases. Nature.

[39] Liu X., Hao W., Qin Y., Decker Y., Wang X., Burkart M., Schötz K., Menger M.D., Fassbender K., Liu Y. (2015). Long-term treatment with Ginkgo biloba extract EGb 761 improves symptoms and pathology in a transgenic mouse model of Alzheimer’s disease. Brain Behav. Immun..

[40] Cho I.-H. (2012). Effects of Panax ginseng in Neurodegenerative Diseases. J. Ginseng Res..

[41] Li W.Z., Li W.P., Zhang W., Yin Y.Y., Sun X.X., Zhou S.S., Xu X.Q., Tao C.R. (2011). Protective Effect of Extract of Astragalus on Learning and Memory Impairments and Neurons Apoptosis Induced by Glucocorticoids in 12-Month Male Mice. Anat. Rec..

[42] Brosnan J.T., Brosnan M.E. (2013). Glutamate: A truly functional amino acid. Amino Acids.

[43] Zhou Y., Danbolt N.C. (2014). Glutamate as a neurotransmitter in the healthy brain. J. Neural Transm..

[44] Lee D.S., Jeong G.S. (2016). Butein provides neuroprotective and anti-neuroinflammatory effects through Nrf2/ARE-dependent haem oxygenase 1 expression by activating the PI3K/Akt pathway. Br. J. Pharmacol..

[45] Sukprasansap M., Chanvorachote P., Tencomnao T. (2017). Cleistocalyx nervosum var. paniala berry fruit protects neurotoxicity against endoplasmic reticulum stress-induced apoptosis. Food Chem. Toxicol..

[46] Prasad K.N. (2016). Simultaneous activation of Nrf2 and elevation of antioxidant compounds for reducing oxidative stress and chronic inflammation in human Alzheimer’s disease. Mech. Ageing Dev..

[47] Liu Z., Zhou T., Ziegler A.C., Dimitrion P., Zuo L. (2017). Oxidative Stress in Neurodegenerative Diseases: From Molecular Mechanisms to Clinical Applications. Oxid. Med. Cell. Longev..

[48] Fujino M., Nishio Y., Ito H., Tanaka T., Li X.-K. (2016). 5-Aminolevulinic acid regulates the inflammatory response and alloimmune reaction. Int. Immunopharmacol..

[49] Loboda A., Damulewicz M., Pyza E., Jozkowicz A., Dulak J. (2016). Role of Nrf2/HO-1 system in development, oxidative stress response and diseases: An evolutionarily conserved mechanism. Cell. Mol. Life Sci..

[50] Paine A., Eiz-Vesper B., Blasczyk R., Immenschuh S. (2010). Signaling to heme oxygenase-1 and its anti-inflammatory therapeutic potential. Biochem. Pharmacol..

